# Assessment of Acute Hospital Use and Transfers for Management of Pediatric Seizures

**DOI:** 10.1001/jamanetworkopen.2020.3148

**Published:** 2020-04-21

**Authors:** Urbano L. França, Michael L. McManus

**Affiliations:** 1 Division of Critical Care, Department of Anesthesiology, Critical Care and Pain Medicine, Boston Children’s Hospital, Boston, Massachusetts; 2 Harvard Medical School, Boston, Massachusetts

## Abstract

**Importance:**

The availability of pediatric hospital care for common conditions is decreasing across the US. The consequences of this decrease on access to care for specific conditions need to be evaluated.

**Objective:**

To evaluate the degree of regionalization of pediatric seizure care in the US by characterizing the activity of hospital systems in 6 diverse states.

**Design, Setting, and Participants:**

This retrospective cross-sectional study used inpatient and emergency department administrative data sets from all acute care hospitals in Arkansas, Florida, Kentucky, Maryland, Massachusetts, and New York from 2014. All patients younger than 18 years who visited a hospital and had a primary diagnosis of seizures were included. Data were analyzed between January and June 2019.

**Main Outcomes and Measures:**

Characteristics of hospital encounters and pediatric Hospital Capability Index scores of transferring and admitting hospitals.

**Results:**

Among 57 930 encounters with pediatric patients with seizures (median [range] age, 4 [1-11] years; 31 968 [55.2%] boys) identified in 621 acute care hospitals, 15 467 patients (26.7%) were admitted as inpatients and 3748 patients (6.5%) were transferred between acute care hospitals. Among encounters that resulted in transfers between hospitals, seizure was the only diagnosis in 1554 patients (41.5%). A total of 42 463 encounters began as emergency department visits, of which 38 173 encounters (90.0%) resulted in routine discharge. While 536 hospitals (86.3%) transferred children with seizures, only 232 hospitals (37.4%) ever admitted them and only 63 hospitals (10.1%) ever received a pediatric seizure transfer. The median (interquartile range) pediatric Hospital Capability Index score of all hospitals was 0.10 (0.02-0.28), while that of hospitals occasionally admitting pediatric seizure patients was 0.34 (0.22-0.55). However, although most patients who were admitted had brief stays (ie, ≤2 days) and no comorbidities, three-quarters of all admissions (12 002 admissions [77.6%]) were to very highly capable centers (ie, hospitals with pediatric Hospital Capability Index scores >0.75). Across all states, the number of referral hospitals for pediatric seizures was less than the number of Dartmouth Atlas Hospital Referral Regions (47 referral hospitals vs 63 hospital referral regions).

**Conclusions and Relevance:**

These findings suggest that although children with seizures are seen in almost all acute care hospital emergency departments, most hospitals transfer children who require admission. Condition-specific interhospital dependency challenges standard definitions of network adequacy and should be accounted for in emergency medical service planning, access to care policies, and health services research.

## Key Points

**Question:**

What is the availability of acute hospital care for pediatric patients with seizures?

**Findings:**

This cross-sectional study including 57 930 encounters with pediatric patients with seizures found that children with seizures are commonly seen in nearly all acute care hospital emergency departments. Most are treated as outpatients, but admission usually requires transfer to a very small number of high-capability receiving hospitals.

**Meaning:**

These findings suggest that pediatric seizure care is highly regionalized; therefore, as health systems and insurance products evolve, adequate networks of pediatric care must include both community hospitals and their quaternary referral partners.

## Teaser

This cross-sectional study examines the regional variation in availability of hospital care for children with seizures.

## Introduction

Pediatric hospital care is consolidating throughout the US, and its availability is decreasing even for common conditions.^1^ This has important implications for access to care, disaster management, and determination of network adequacy. As part of this process, an informal network of interhospital transfer has arisen to compensate for the diminishing capabilities of individual institutions.^2^ However, little is known about the structure, nature, and condition-specific functioning of these networks.

Meanwhile, in response to increasing financial pressure, many insurers seek to control their costs through the creation of care networks. Under these arrangements, health care systems are included within networks when they agree on compensation rates and are excluded when they do not. Care within the network is then fully covered, while care outside is not. As fewer practitioners accept lower rates, networks narrow and risk exclusion of services, particularly services for children.^3,4^ Regulatory oversight of this process is shared by state and federal agencies, but present quantitative standards for network adequacy are limited for adults and inapplicable to children.^5^

Potential exists for disparity among networks of hospital care that have formed organically through medical practice and insurance networks that form and dissolve through contracting. Therefore, network adequacy determination requires a clear understanding of actual care practice to ensure that contracted networks provide access to care. Since actual practice may dictate that some conditions require transfer for care while others do not, condition-specific understanding and definition of network adequacy is required.

Seizures are extremely common among children and frequently lead to emergency department (ED) visits and hospital admission. An estimated 470 000 US children have active epilepsy,^6^ and in 2015, nervous system disorders were the third leading cause of pediatric ED visits.^7^ Despite this frequency, the pediatric neurology workforce is limited^8^ and access to pediatric neurology specialists is constrained,^4^ so it follows that acute seizure care might organically regionalize. Based on our clinical experience, we hypothesized that acute pediatric seizure care is now very highly regionalized in the US and dependent on a small subset of high-capability centers. To test this hypothesis, describe the hospital care network for seizures, and inform network adequacy definitions for children, we studied ED visits, hospital admissions, and transfer patterns among hospitals treating children with seizures across 6 US states.

## Methods

The data sets used in this study contain no personally identifiable health information and are made available to facilitate public health, policy, and biomedical research; therefore, a waiver of informed consent was obtained from the Boston Children’s Hospital Committee on Clinical Investigation, which approved this study. This study followed the Strengthening the Reporting of Observational Studies in Epidemiology (STROBE) reporting guideline.

### Data Sources

We used State Inpatient^9^ and Emergency Department^10^ databases from Healthcare Cost and Utilization Project (HCUP), along with acute hospital case mix data from the Massachusetts Center for Health Information and Analysis,^11^ to identify all non-newborn children younger than 18 years who presented to hospitals in Arkansas, Florida, Kentucky, Massachusetts, Maryland, and New York during 2014 with a primary HCUP Clinical Classification Software diagnosis of “epilepsy; convulsions” (Clinical Classification Software code 83; a full list of corresponding *International Classification of Diseases, Ninth Revision,*^12^ codes is included in the eAppendix in the Supplement). These 6 states were chosen for their representative mix of size, rurality, population density, and academic medical dominance.

### Statistical Analysis

An encounter was defined as an ED visit or hospital admission, independently of the source of the visit and its eventual disposition.^9,10^ Under this definition, patients transferred among institutions or who had hospital encounters on more than 1 occasion can be present more than once in this cohort. The site, source, outcome, and specifics of each encounter were extracted and summarized separately for each state. Encounter characteristics associated with transfers were evaluated using the disposition or source of admission fields in each encounter.^13^ Although source of admission was not available for most New York encounters, 483 transfers could be identified, and complete information was available in all other states.

Transferring and receiving hospitals were compared using the pediatric Hospital Capability Index (pHCI), and states were compared using the pediatric Regionalization Index (RI). The Hospital Capability Index and RI are metrics that summarize individual hospital and statewide transfer behaviors.^1,2,14^ Hospitals with Hospital Capability Index scores near 1 treat most conditions and seldom transfer patients for a higher level of care, while those with lower Hospital Capability Index scores treat fewer conditions and transfer more frequently. Similarly, states or regions with RI scores closer to 1 are highly regionalized (ie, patients receive definitive care in just a few institutions) and those with RI scores closer to 0 are less regionalized (ie, definitive care is available in more places). As described elsewhere,^14^ both variables may be straightforwardly stratified by condition or by variables of interest, such as age, sex, or insurance status. Here, we report pediatric Hospital Capability and Regionalization Indices calculated overall and condition-specific for seizures. All calculations were conducted separately for each state using complete HCUP and Center for Health Information and Analysis data sets. Encounter rates were based on 2014 US Census Bureau population estimates^15^ and calculated as the number of encounters in 2014 for every 1000 residents younger than 18 years.

For comparison of pediatric seizure care regionalization with regionalization of adult care, 2014 Medicare-based hospital service areas (HSAs) and hospital referral regions (HRRs) were obtained from the Dartmouth Atlas.^16^ The Dartmouth Atlas Project uses large claims databases from the Centers for Medicare & Medicaid Services to define HSAs as the collection of zip codes whose Medicare residents receive most of their hospitalizations from hospitals in that area. As some technical services are highly regionalized and available only in large referral centers, HRRs were created to represent regional health care markets for tertiary and quaternary medical care, such as major cardiovascular or neurosurgical procedures.

Length of stay, ages of different cohorts, and hospital capabilities of groups of hospitals were compared using Mann-Whitney *U* tests. All analyses were conducted using open source data science tools within Jupyter notebooks running Python 3.6.^17^ *P* values were 2-tailed, and statistical significance was set at *P* < .05. Data were analyzed between January and June 2019.

## Results

### Hospital Encounters

A total of 57 930 encounters with pediatric patients with seizures (median [range] age, 4 [1-11] years; 31 968 [55.2%] boys) were identified in 621 acute care hospitals. Demographic characteristics are presented in Table 1. In 22 313 encounters (38.5%), the primary diagnosis of seizure was the only diagnosis assigned, and in 7651 encounters (13.2%), there were 5 or more diagnoses. Among all encounters, 15 467 (26.7%) were admitted as inpatients and 3748 (6.5%) resulted in transfer, including 3609 transfers (6.2%) from EDs and 139 transfers (0.2%) from inpatient settings. Among transfers, seizure was the only assigned diagnosis in 1554 transfers (41.5%), and 5 or more diagnoses were present in 349 transfers (9.3%).

**Table 1.** Demographic Characteristics of Children With Seizures Presenting at Hospitals

Characteristic	Encounter type, No. (%)			
	All (N = 57 930)	ED visits (n = 42 463)	Admissions (n = 15 467)	Transfers (n = 3748)
Age, median (IQR), y	4 (1-11)	4 (1-11)	5 (2-11)	4 (1-10)
Sex				
Boys	31 968 (55.2)	23 544 (55.4)	8424 (54.5)	2009 (53.6)
Girls	25 960 (44.8)	18 917 (44.6)	7043 (45.5)	1738 (46.4)
Race/ethnicity				
Black	13 117 (22.6)	10 236 (24.1)	2881 (18.6)	699 (18.6)
Hispanic	8.802 (15.2)	6208 (14.6)	2594 (16.8)	514 (13.7)
White	23 419 (40.4)	17 442 (41.1)	5977 (38.6)	1724 (46.0)
Other or unknown	12 592 (21.7)	8577 (20.2)	4015 (26.0)	811 (21.6)

Abbreviations: ED, emergency department; IQR, interquartile range.

Most ED visits resulted in completion of care. Of 42 463 ED encounters, 38 173 (90.0%) resulted in routine discharge, 404 (0.9%) resulted in return to an intermediate care facility, and 231 (0.5%) resulted in elopement. Transfer from an ED to a higher level of care was coded after 3609 ED encounters (8.5%). There were fewer than 11 ED deaths in total.

Among transferred patients, most received routine seizure care. Of 1826 encounters coded as received transfers from acute care hospitals, 227 (12.4%) were discharged from the receiving EDs. Patients admitted after transfer were slightly younger than other admissions (median [IQR] age, 4 [1-10] years vs 5 [2-11] years; *P* < .001) but did not differ significantly in median (IQR) length of stay (2 [1-3] days for both; *P* = .59).

### Hospitals

In each state, nearly all hospitals saw children with seizures, but only some hospitals admitted them for care. In all, 621 hospitals encountered pediatric patients with seizures, with 232 hospitals (37.4%) admitting them and 536 hospitals (86.3%) transferring them. Half of all ED routine discharges occurred in 545 hospitals (87.8%) with pHCI scores less than 0.50. Only 63 hospitals (10.1%) reported ever receiving a patient with seizures in transfer and approximately one-quarter of these (15 hospitals [23.8%]) received only 1 or 2 patients. Because the median (IQR) pHCI score was very high for hospitals receiving more than 2 transfers (0.86 [0.75-0.91]) and was modest for hospitals receiving 2 or fewer transfers (0.37 [0.30-0.51]), these may represent retrotransfer patients returning from referral centers to complete care closer to home. For this reason, the number of hospitals in each state receiving more than 2 patients with seizures is reported in Table 2.

**Table 2.** Interhospital Transfer of Children With Seizures by State in 2014

Characteristic	No.						
	Arkansas	Florida	Kentucky	Massachusetts	Maryland	New York	Total
Hospitals	72	184	98	67	46	167	634
Encountering pediatric seizures	70	179	97	64	46	165	621
Admitting pediatric seizures	22	61	31	33	18	67	232
Transferring pediatric seizures	58	154	84	58	43	139	536
Receiving >2 pediatric seizure transfers	<3^a^	21	<3^a^	5	3	15	47
Pediatric RI (seizure-specific pediatric RI)	0.58 (0.88)	0.56 (0.81)	0.66 (0.88)	0.55 (0.78)	0.59 (0.86)	0.43 (0.66)	NA
Dartmouth Atlas							
HSAs	61	110	79	49	30	144	473
HRRs	7	19	9	5	6	17	63

Abbreviations: HRR, hospital referral region; HSA, hospital service area; NA, not applicable; RI, Regionalization Index.

^a^ Owing to data use agreement reasons, we cannot identify fewer than 2 hospitals per cell.

Most pediatric seizure admissions (12 002 admissions [77.6%]) went to very high–capability hospitals (ie, hospitals with pediatric Hospital Capability Index scores >0.75). Although the median (IQR) pHCI score of all hospitals ever seeing children with seizures was 0.10 [0.02-0.28], the median (IQR) pHCI score of all hospitals that ever admitted children with seizures was 0.34 [0.22-0.55] (*P* < .001). Moreover, more than 75% of all admissions were reported by just 38 hospitals (6.1%) that were extremely capable (pHCI score >0.75). For comparison, the sample of all admitting hospitals contained several freestanding children’s hospitals, with pHCI scores ranging from 0.78 to 0.91. The volumes and distributions of patients seen, transferred, and admitted to hospitals of varying capability are presented in the Figure.

**Figure.**
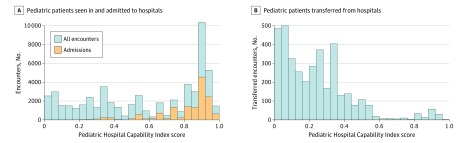
Dispositions of Pediatric Patients With Seizures Presenting at Hospitals A, Number of pediatric seizure patients seen in (blue) and admitted to (orange) hospitals of various capabilities. B, All pediatric patients with seizures transferred from hospitals of various capabilities.

### State-Specific Networks of Care

Over all 6 states, there were 4.40 encounters per year for every 1000 residents younger than 18 years. Examination of individual states revealed similar encounter rates and age distributions but differing admission and transfer rates (Table 3). As shown in Table 2, regionalization of care for pediatric seizures was much higher than for general pediatric care in each state. Although the number of receiving hospitals correlated strongly with population (Pearson *r* = 0.95), there was no correlation with geographic size, and there was considerable variability among states in the number of receiving hospitals per million children (range, 2.15-6.41). In all states, the number of Dartmouth Atlas HSAs exceeded that of hospitals ever admitting pediatric patients with seizure (Arkansas: 61 HSAs vs 22 admitting hospitals; Florida: 110 HSAs vs 61 admitting hospitals; Kentucky: 79 HSAs vs 31 admitting hospitals; Massachusetts: 49 HSAs vs 33 admitting hospitals; Maryland: 30 HSAs vs 18 admitting hospitals; New York: 144 HSAs vs 67 admitting hospitals). In 4 states, there were fewer hospitals receiving transfers of pediatric patients with seizures than there were adult tertiary health care markets (ie, Dartmouth Atlas HRRs) (overall: 47 referral hospitals vs 63 HRRs; Arkansas: <3 receiving hospitals vs 7 HRRs; Kentucky: <3 receiving hospitals vs 9 HRRs; Maryland: 3 receiving hospitals vs 6 HRRs; New York: 15 receiving hospitals vs 17 HRRs) (Table 2).

**Table 3.** Hospital Encounters, Admissions, and Transfers in 2014

Characteristic	No.					
	Arkansas	Florida	Kentucky	Massachusetts	Maryland	New York
Encounters	2933	19 372	4644	6233	5005	19 743
Rate per 1000 children	3.98	4.60	4.48	4.34	3.58	4.53
Admissions	487	4784	762	1826	525	7083
Rate, %	16.6	24.7	16.4	29.3	10.5	35.9
Transfers	270	1285	457	524	497	715
Rate, %	9.2	6.6	9.8	8.4	9.9	3.6

## Discussion

The findings of this cross-sectional study suggest that hospital care for pediatric seizures is extremely regionalized and very highly dependent on a system of interhospital transfer. In clinical practice, children with seizures present to nearly all acute care hospitals, but only a subset of hospitals ever admit them. While most children are treated and released from the ED, many require additional care, which is often available only through transfer. Most transfers are to very high–capability centers, but transferred patients usually have few (or no) additional diagnoses and are often discharged directly from the receiving ED, or, if admitted, experience short stays.

This study adds to the growing literature on access to pediatric care and focuses on a routine pediatric neurological condition. In this study, the high rate of routine home discharge suggests that good first-line care is available in most hospitals. However, transfer activity suggests that there is frequent need for additional expertise and services that are available only in a small number of specialized referral centers. Transfers discharged directly from receiving EDs may have required imaging or a specialist’s assessment. Short admissions may have required these as well as a brief period of expert observation under therapy. While formal pediatric network adequacy metrics are lacking and adult metrics are inappropriate,^5^ our findings suggest that any adequate network for seizure care should include both community hospitals (where successful emergency care is frequently provided) and their referral partners (where definitive care can be obtained when necessary). Limiting the former may bring distance barriers and delays, while limiting the latter may lead to inadequate care or inappropriate out-of-network costs.

Condition-specific understanding of hospital interrelatedness is also relevant to facility designation and emergency medical services point-of-entry policies. In most regions of the country, formal destination protocols are lacking for pediatric patients presenting with non–trauma-related conditions, but emergency medical services clinicians, aware of hospital differences, elect to bypass the nearest facilities in nearly half of their pediatric transports.^18^ Although pediatric readiness is usually focused on ED care,^19^ our findings suggest that inpatient capabilities and the likelihood of secondary transport should be considered as well.

Hospital service areas and HRRs as presented in the Dartmouth Atlas^16^ have been widely used to study regional variations in health care.^20^ However, our examination of seizure care is consistent with other work indicating that Medicare-based HSAs and HRRs are of limited value in pediatrics.^21^ This is because children experience a very different system of care,^1,21^ necessitating separate formulations of service areas.^22^ Our observations suggest that effective HSAs for a relatively common pediatric condition, such as seizures, requires inclusion of a transfer-receiving hospital. If so, pediatric seizure care must be considered at least as regionalized as the adult neurosurgical and cardiovascular care by which the Dartmouth HRR’s are defined. More accurate and valid representation of pediatric HSAs will require additional exploration of condition-specific transfer patterns.

Finally, the findings of this study suggest that significant opportunity for increased interhospital collaboration exists. For example, regular transfer partners might standardize their ED practices and streamline information transfer. When distances are long or capacity is limited, they could explore telemedicine to facilitate safe treatment without transfer. If lengths of stay warrant and care needs permit, retrotransfer options could be considered. Finally, when accepting centers are persistently crowded, capabilities among referral hospitals could be increased.

### Limitations

This study has several limitations. It is subject to all of the limitations that accompany retrospective studies using large administrative data sets.^23^ More specifically, while all transfers out of hospitals can be reliably identified in disposition fields, there are 3 potential sources of error when attempting to identify receiving hospitals from source of admission fields. First, transfer status could be missed when source of admission is not recorded. Although this was often the case in New York, nearly 500 transfers were available for study, and we believe it unlikely that a major in-state referral center was missed. Second, because HCUP databases are state-based, we were unable to detect cross-border transfers to contiguous states. Geographically, the magnitude of this consideration would be expected to vary from state to state, but our general findings did not. Finally, among hospitals receiving very few transfers, we were unable to definitively distinguish those representing transfer for a higher level of care from those returning to their community from tertiary centers. For comparison with HRRs, we elected to conservatively define receiving hospitals as any institution reporting receipt of more than 2 transfers. Although this likely overestimates the true availability of definitive seizure care, we believe it provides an upper bound.

## Conclusions

This cross-sectional study found that hospital care for pediatric patients with seizures was highly regionalized and admissions were largely concentrated within a small number of high-capability referral centers. This was accomplished through an informal network of interhospital transfer that should be accounted for in health policy, determinations of network adequacy, emergency medical service planning, and health services research.

## Notes

Supplement. **eAppendix.** Supplementary Methods
