# Supplementary material for: Assessment of Acute Hospital Use and Transfers for Management of Pediatric Seizures
Source: JAMA Netw Open. 2020 Apr 21;3(4):e203148. doi: 10.1001/jamanetworkopen.2020.3148 (PMC7175083; doi:10.1001/jamanetworkopen.2020.3148)
Supplement: Supplement. — eAppendix. Supplementary Methods [file jamanetwopen-3-e203148-s001.pdf]

Supplementary Online Content

França UL, McManus ML. Assessment of acute hospital use and transfers for management of pediatric seizures. *JAMA Netw Open*. 2020;3(4):e203148. doi:10.1001/jamanetworkopen.2020.3148

eAppendix. Supplementary Methods

This supplementary material has been provided by the authors to give readers additional information about their work.

© 2019 França UL et al. *JAMA Network Open*.

eAppendix. Supplementary Methods

Hospital Capability and Regionalization Indices

We provide in this appendix a brief overview of both the pediatric Hospital Capability Indice (pHCI) and the pediatric Regionalization Indices (pRI) discussed in the manuscript. A more complete discussion of the theoretical underpinnings can be found at França & McManus (2017).[1] These indices are based upon the *probability of care completion (P),* defined as the ratio of the number admissions to the sum of admissions *(A) and transfers (T) at a given* hospital:

$$\mathcal{P}(\mathrm{h}, \mathrm{CCS}, \mathrm{time}, \mathrm{age}) = \frac{A(\mathrm{h}, \mathrm{CCS}, \mathrm{time}, \mathrm{age})}{A(\mathrm{h}, \mathrm{CCS}, \mathrm{time}, \mathrm{age}) + T(\mathrm{h}, \mathrm{CCS}, \mathrm{time}, \mathrm{age})}$$

The probability of care completion above is defined by hospital (*h*), period of time, and CCS code, which give the *minimal* set of variables, but additional ones, such as age, insurance, etc., could in principle be used to stratify the population. Using this probability of care completion allows us to define a pediatric Hospital Capability Index (pHCI) used in the manuscript,

$$\mathrm{pHCI}(\mathrm{h},\ 2014, \mathrm{age} < 18) = \frac{\sum_{\mathrm{CCS}} \mathcal{P}(\mathrm{h}, \mathrm{CCS},\ 2014, \mathrm{age} < 18)}{\mathrm{Number\ of\ CCS\ codes}(2014, \mathrm{age} < 18)}$$

where the denominator consists on all CCS codes observed in 2014 in each state for this population. Similarly, we can define the condition-specific pediatric Regionalization Index (condition-specific pRI) in each state as,

$$\mathrm{pRI}(\mathrm{CCS}, 2014, \mathrm{age} < 18) = 1 - \frac{\sum_{\mathrm{h}} \mathcal{P}(\mathrm{h}, \mathrm{CCS},\ 2014, \mathrm{age} < 18)}{\mathrm{Number\ of\ hospitals}(2014)}$$

where the denominator corresponds to the total number of acute care hospitals in the state in 2014. In the manuscript, we define pRIsz = pRI(CCS=83: *Epilepsy; convulsions*, 2014, age < 18). Finally, we can define the average pRI for each state in a 2014 as

$$\mathrm{pRI}(2014, \mathrm{age} < 18) = \frac{\sum_{\mathrm{CCS}} \mathrm{pRI}(\mathrm{CCS}, 2014, \mathrm{age} < 18)}{\mathrm{Number\ of\ CCS\ codes}(2014, \mathrm{age} < 18)}$$

which describes the average regionalization of all pediatric conditions in the state in 2014.

© 2019 França UL et al. *JAMA Network Open*.

List of International Disease Classification, Ninth Revision (ICD-9-CM) codes

List of ICD-9-CM codes encompassed by the Clinical Classification Software (CCS) single-level diagnosis code 83, "*Epilepsy; convulsions*" (for details, see Ref. 3).

ICD-9-CM CODE	ICD-9-CM CODE DESCRIPTION
345.0	Generalized nonconvulsive epilepsy
345.00	Generalized nonconvulsive epilepsy, without mention of intractable epilepsy
345.01	Generalized nonconvulsive epilepsy, with intractable epilepsy
345.1	Generalized convulsive epilepsy
345.10	Generalized convulsive epilepsy, without mention of intractable epilepsy
345.11	Generalized convulsive epilepsy, with intractable epilepsy
345.2	Petit mal status
345.3	Grand mal status
345.4	Localization-related (focal) (partial) epilepsy and epileptic syndromes with complex partial seizures
345.40	Localization-related (focal) (partial) epilepsy and epileptic syndromes with complex partial seizures, without mention of intractable epilepsy
345.41	Localization-related (focal) (partial) epilepsy and epileptic syndromes with complex partial seizures, with intractable epilepsy
345.5	Localization-related (focal) (partial) epilepsy and epileptic syndromes with simple partial seizures
345.50	Localization-related (focal) (partial) epilepsy and epileptic syndromes with simple partial seizures, without mention of intractable epilepsy
345.51	Localization-related (focal) (partial) epilepsy and epileptic syndromes with simple partial seizures, with intractable epilepsy
345.6	Infantile spasms
345.60	Infantile spasms, without mention of intractable epilepsy
345.61	Infantile spasms, with intractable epilepsy
345.7	Epilepsia partialis continua
345.70	Epilepsia partialis continua, without mention of intractable epilepsy
345.71	Epilepsia partialis continua, with intractable epilepsy
345.8	Other forms of epilepsy and recurrent seizures
345.80	Other forms of epilepsy and recurrent seizures, without mention of intractable epilepsy
345.81	Other forms of epilepsy and recurrent seizures, with intractable epilepsy
345.9	Epilepsy, unspecified
345.90	Epilepsy, unspecified, without mention of intractable epilepsy
345.91	Epilepsy, unspecified, with intractable epilepsy
780.3	Convulsions
780.31	Febrile convulsions (simple), unspecified
780.32	Complex febrile convulsions
780.33	Post traumatic seizures
780.39	Other convulsions

© 2019 França UL et al. *JAMA Network Open*.

References
1. França UL, McManus ML. Transfer Frequency as a Measure of Hospital Capability and Regionalization. *Health Services Research* 2017;52(6):2237–2255. doi:10.1111/1475-6773.12583.

2. França UL, McManus ML. Availability of Definitive Hospital Care for Children. *JAMA Pediatr*. 2017;171(9):e171096. doi:10.1001/jamapediatrics.2017.1096.

3. Healthcare Cost and Utilization Project. Clinical classifications software (CCS) for ICD-9-CM, 2006-2009. https://www.hcup-us.ahrq.gov/toolssoftware/ccs/ccs.jsp; 2013.

© 2019 França UL et al. *JAMA Network Open*.
